# Molecular response to induction chemotherapy and its correlation with treatment outcome in head and neck cancer patients by means of NMR-based metabolomics

**DOI:** 10.1186/s12885-021-08137-4

**Published:** 2021-04-15

**Authors:** Łukasz Boguszewicz, Agata Bieleń, Jarosław Dawid Jarczewski, Mateusz Ciszek, Agnieszka Skorupa, Krzysztof Składowski, Maria Sokół

**Affiliations:** 1 Department of Medical Physics, Maria Sklodowska-Curie National Research Institute of Oncology, Gliwice Branch, Warszawa, Poland; 2 1st Radiation and Clinical Oncology Department, Maria Sklodowska-Curie National Research Institute of Oncology, Gliwice Branch, Warszawa, Poland; 3 Radiology and Diagnostic Imaging Department, Maria Sklodowska-Curie National Research Institute of Oncology, Gliwice Branch, Warszawa, Poland

**Keywords:** NMR, Metabolomics, Chemotherapy, Head and neck, Treatment outcome

## Abstract

**Background:**

The aim of this prospective study is to identify the biomarkers associated with the effects of induction chemotherapy (iCHT) in terms of the favorable/weaker response to the treatment in locally advanced head and neck squamous cells carcinomas (LA-HNSCC).

**Methods:**

The studied group consisted of 53 LA-HNSCC patients treated with iCHT. The treatment tolerance was measured by the Common Terminology Criteria for Adverse Events (CTCAE). The response to the treatment was evaluated by the clinical, fiberoptic and radiological examinations made before and after iCHT (the TNM Classification of Malignant Tumors was used for classifying the extent of cancer spread). Proton nuclear magnetic resonance (^1^H NMR) serum spectra of the samples collected before and after iCHT were acquired with a 400 MHz spectrometer and analyzed using the multivariate and univariate statistical methods.

**Results:**

The molecular response to iCHT involves an increase of the serum lipids which is accompanied by the simultaneous decrease of alanine, glucose and N-acetyl-glycoprotein (NAG). Furthermore, in males, the iCHT induced changes in the lipid signals and NAG significantly correlate with the regression of the primary tumor. The OPLS-DA multivariate model identified two subgroups of the patients with a weaker metabolic and clinical response. The first one consisted of the patients with a significantly lower initial nodal stage, the second one showed no differences in the initial clinical and metabolic statuses.

**Conclusions:**

The NMR-based metabolomic study of the serum spectra revealed that iCHT induces the marked changes in the LA-HNSCC patients’ metabolic profiles and makes it possible to stratify the patients according to their response to iCHT. These effects are sex dependent. Further studies on a larger scale accounting for sex and the clinical and metabolic factors are warranted.

**Supplementary Information:**

The online version contains supplementary material available at 10.1186/s12885-021-08137-4.

## Background

iCHT in the treatment of LA-HNSCC [1–3] is based on two standard regimens, which are the TPF schedule (docetaxel, cisplatin and 5-fluorouracil) and the PF schedule (cisplatin and 5-fluorouracil). TPF demonstrates a less favorable toxicity profile and a better response rate [4, 5]. Severe hematological adverse events (e.g. leukopenia, neutropenia, anemia) are more often reported in TPF [6–8], while non-hematological adverse events (e.g. mucositis, nausea, vomiting) seem to be higher in PF [6–9]. It is suggested that sex is among other factors, like age, performance status, disease status, lesion size, medication exposure and comorbidities, that may contribute, via the genetic, hormonal and molecular pathways, to the chemotherapy efficacy and its toxicity [10, 11].

Despite the improvements in treatment and new therapies, the survival rates in LA-HNSCC remain stagnant [12–14]. Thus, there is the urgent need for novel approaches that can be used to monitor both the tolerability and the treatment efficacy. Especially body fluid and liquid biopsy biomarkers appear to hold a strong potential for development the tools for such applications that could direct anticancer therapies in HNSCC [15]. In recent years, there is an increasing number of the reports based on the metabolomic approaches attempting to monitor chemotherapy side effects and its efficacy in various cancer types [16–21]. In head and neck cancers Ye at al. revealed the important metabolic differences in the iCHT efficacy between the responsive and unresponsive patients using the chromatographic methods [21]. They compared the pre- and post-chemotherapy serum samples and found that induction chemotherapy induces various metabolic changes, e.g. in carbohydrate metabolism, amino acid metabolism, fatty acid metabolism and steroid metabolism. Lactic acid, glutamic acid, and aspartic acid were even defined as the potential biomarkers of the iCHT efficacy [21]. On the other hand, Jelonek et al. [22] analyzed the chromatographic profiles of the sera of the HNSCC patients treated with concurrent chemo-radiotherapy or radiotherapy alone with or without prior induction chemotherapy. They detected the differences in the molecular responses to the various treatment modalities, hypothetically associated with a different risk of toxicity, however no metabolic abundance changes between the pre- and post-treatment samples in case of iCHT were observed, presumably due to restricting their study only to the first iCHT cycle.

In the current work we decided to perform the NMR-based study of the influence of iCHT on the serum metabolome of the LA-HNSCC patients to identify the NMR biomarkers of the iCHT-induced metabolic changes and to assess their relationships with the response to treatment.

It may be hypothesized that such NMR approach may help to differentiate the well responding patients from the nonresponding ones, to explain the causes of the metabolic differences and to determine the influence of sex on the therapy response.

## Methods

### Characteristics of patient groups

The study was approved by Ethical Committee of the Maria Sklodowska-Curie National Research Institute of Oncology, and written informed consent was obtained from each patient. The retrospective study was conducted on the HNSCC patients treated oncologically in the 1st Radiation and Clinical Oncology Department of Maria Sklodowska-Curie National Research Institute of Oncology, Gliwice Branch. The study group consisted of 35 men and 18 women, all Caucasians, at the median age of 57 (22–74) years. Induction chemotherapy is mainly used in the advanced cancer stages (LA-HNSCC), therefore the patients in the current study where staged to TNM IV (81.1%) and III (18.9%). There were no patients with metastases (M0 = 100%), all patients were HPV negative.

The protocols (regimens) of iCHT were as follows:

- 3 cycles of TPF administered every 21 days (docetaxel 75 mg/m^2^, followed by cisplatin 100 mg/m2 on day 1, and 5-fluorouracil 1000 mg/m^2^ per day administered as a continuous 24-h infusion for 4 days) followed by chemoradiotherapy delivered as a sequential therapy - 21 patients [4]

or

b) 4 cycles of TPF administered every 21 days (docetaxel 75 mg/m^2^ of body-surface area, followed by cisplatin 75 mg/m^2^ on day 1, 5-fluorouracil 750 mg/m^2^ per day administered as a continuous 24-h infusion for 4 days) followed by radiotherapy as a sequential therapy - 2 patients [5]

or

c) 3 cycles of PF administered every 21 days (cisplatin 100 mg/m^2^ on day 1, 5-fluorouracil 1000 mg/m^2^ as a continuous 24-h infusion for 4 days) followed by chemoradiotherapy with cisplatin delivered as a sequential therapy – 28 patients [4, 23]

or

d) 3 cycles of paclitaxel and carboplatin (PC) administered every 21 days (175 mg/m^2^) and carboplatin at a dose calculated using the Calvert formula area under the curve of 5 followed by chemoradiotherapy with carboplatin delivered as a sequential therapy – 2 patients [24].

Induction chemotherapy was administered every 3 weeks (defined as one cycle) for up to four cycles, unless progressive disease, unacceptable toxic effects, or withdrawal from the study occurred earlier. The patients received one (9 patients), two (6 patients), three (36 patients) or four iCHT cycles (2 patients). The patients with an incomplete iCHT were treated with the TPF (7 patients) and PF (8 patients) regimens. The administration of the antiemetics, antibiotics, steroids, hydration, diuretics was mandatory during each cycle.

In aim of quantifying the patients’ general well-being and the activities of daily life their performance status was measured to determine whether they can receive chemotherapy. All of them had 2 or less performance status by WHO/ECOG/Zubrod scale, the adequate hematologic, renal, and hepatic functions, without serious comorbidities and was able to receive iCHT. During chemotherapy, the patients were monitored clinically and with the laboratory tests on day 1 of each cycle, before starting the treatment. The iCHT timing depended on the treatment tolerance measured by the Common Terminology Criteria for Adverse Events (CTCAE) - the standardized classification of adverse effects of cancer therapy [25]. CTCAE uses a range of grades from 1 to 5 (1 – Mild, 2 – Moderate. 3 – Severe, 4 - Life-threatening, 5 – Death). The most common causes of the delays in cycles’ administration were neutropenia or/and leukopenia grade 3 and more.

### Assessment of treatment response to induction chemotherapy

The treatment response to induction chemotherapy was evaluated by the clinical, fiberoptic and radiological examinations. The TNM Classification of Malignant Tumors v. 7 was used before and after iCHT as a globally recognized standard for classifying the extent of cancer spread (T - tumor, N – regional involved lymph nodes, M - distant metastasis). The prefix modifiers were used as follows: “c” – a stage determined from the evidence acquired before the treatment (including the clinical examination, imaging, endoscopy, biopsy, surgical exploration), “y” – a stage assessed after iCHT in the same way as “c”. In our study most of the patients were the significant responders (yTNM lower than cTNM and an explicit percentage volumetric radiological remission) and iCHT was followed by radiotherapy or concurrent chemoradiotherapy in compliance with a schedule.

The primary lesions were measured within the head and neck in the images taken before (preCHT) and after induction chemotherapy (postCHT) in the radiology department of the Maria Sklodowska-Curie National Research Institute of Oncology. If an incomplete cycle of induction chemotherapy and immediate subsequent radical radiochemotherapy was performed (7 patients), the CT scan for the radiotherapy planning was used after induction chemotherapy for a sake of comparison. All types of imaging capable of obtaining a multiplanar reconstruction (MPR) were considered (CT, MR and PET-CT scans).

All volumetric measurements were performed by a qualified radiologist using the ‘MR Segmentation’ tool which is available with Siemens Healthcare syngo.via software, version VB20A. The following procedure was applied for each lesion. After the image was viewed with the ‘MR Basic’ system, the ‘MPR view’ was enabled. Then the ‘MR Segmentation tool’ was selected from the ‘VOI selection’ list to evaluate an arbitrary VOI in a semiautomatic way. The segmentation was performed using the ‘brush based segmentation’ tool. The middle part of the lesion was marked by tracing it with a green line, its boundary was enclosed by tracing it with a red line. The lesion area was defined in this way on every third layer. The starting and ending layers of the volume segmentation were marked with a solid red area only. Finally, the lesion volume was calculated, measured in cubic centimeters, and limited by a green outline.

The percentage of tumor regression was calculated using the following formula: 100-(postCHT tumor volume)*100/(preCHT tumor volume). The clinical response to iCHT was expressed as a difference of cT-yT and cN-yN.

### Serum samples collection

The overnight fasting blood samples from the peripheral vein were collected before (preCHT) the first cycle of iCHT and within a week preceding the start of radiation therapy (postCHT), at the median of 45 days after the end of iCHT. The blood samples were incubated for 30 min at room temperature and then centrifuged (1000×g, 10 min) to remove a clot, and stored frozen at − 80 °C until the NMR measurements are performed. The total number of 130 samples were collected.

### Sample preparation for NMR spectroscopy

The serum samples were thawed in two steps (at 4 °C and at room temperature) and mixed with the phosphate buffer (pH 7.4) containing D_2_O and TSP. The aliquots of 600 μl of the solution were transferred into 5 mm Wilmad WG-1235-7 NMR tubes (Wilmad Labglass, USA) and kept at 4 °C until the NMR analysis.

### Measurement protocol

The same measurement protocol as in our previous metabolomic studies [26, 27] was applied. The ^1^H NMR spectra were acquired on a Bruker 400 MHz Avance III spectrometer (Bruker Biospin, Rheinstetten, Germany) equipped with a 5 mm PABBI probe. The quality control tests were performed at every measurement day. The NMR probe tuning and matching, shimming, determination of the transmitter offset value for the water pulse presaturation and 90° pulse adjustments were always made for each sample. The receiver gain was set to 90.5 and the temperature to 310 K for all experiments. Four different ^1^H NMR spectra – NOESY (1D nuclear Overhauser enhancement spectroscopy), CPMG (Carr-Purcell-Meiboom-Gill), diffusion edited (DIFF) and J-resolved (JRES) – were acquired for each serum sample. The characteristics of the acquired spectra as well as the pulse sequence parameters are given in Supplementary Material (Table S1).

### Spectra post-processing

All 1D spectra were processed with a line broadening of 0.3 Hz and automatically phase corrected (in Topspin software from Bruker Biospin), referenced to the methyl doublet of alanine at 1.5 ppm and bucketed over the region 9.0–0.5 ppm with the bucket width set to 0.002 ppm using AMIX software (Bruker Biospin). The spectrum region of water (5.15–4.38 ppm, d = 0.77 ppm) was removed from the analysis in order to prevent variation in each sample. No normalization was applied. This is the standard processing protocol used in our metabolomic lab [26, 27].

### Metabolites identification

The metabolites identification was done based on the comparisons with the reference compounds library (in Chenomx NMR Suite Professional (Chenomx Inc., Edmonton, Canada)), as well as on the multiplicity and scalar couplings information extracted from the 2D JRES spectra, and using the information from Human Metabolome Database (http://www.hmdb.ca/) and the available literature.

### Metabolite quantification

The low molecular weight metabolites were quantified based on the 1D positive projections of the JRES spectra. The diffusion edited spectra were used for quantification of the lipid signals. The integrals were measured in the spectral regions defined individually for each metabolite using the “sum all points in region” method in AMIX (Bruker Biospin) software.

### Data analysis and validation of multivariate model

The data analysis was carried out using SIMCA-P+ (Umetrics, v. 14) software. The post-processed NMR spectra were Pareto scaled and the distinction between the preCHT and postCHT groups was done using the orthogonal partial least squares discriminant analysis (OPLS-DA). The OPLS-DA results are presented graphically in two types of plots, which detailed description and interpretation is available in [26]. The validation of the OPLS-DA model was carried out using the internal cross-validation.

The statistical significance of the estimated predictive power of the OPLS-DA model was tested using permutation testing and ANOVA of the cross-validated residuals (cv-ANOVA) as well as the external test set consisted of 24 serum samples acquired from 24 (LA-HNSCC) patients. The blood samples were collected after iCHT (23 patients) and in case of one patient only the pre-treatment sample was included (the post-treatment one was excluded due to an insufficient blood sample volume). The detailed description of the external test group is given in Supplementary Material (Table S2).

The metabolites’ integrals were evaluated for their statistical significance with the Wilcoxon signed-rank (WSR), Mann-Whitney U (MWU), Kruskal-Wallis ANOVA (KWA) and Spearman’s rank correlations (SRC) tests. The significance threshold was set at 0.05. Statistica software (Statsoft, v. 12) was applied for the univariate statistics.

## Results

The detailed baseline characteristics of the patients under the investigation is presented in Table 1.

**Table 1:** Characteristics of the study group

Baseline patient characteristics of the main studied group			
		No.	%
Age, years	Median	57	
	Range	22–74	
Sex	Male	35	66
	Female	18	34
Primary tumor site	Oropharynx	20	37.7
	Nasopharynx	12	22.6
	Hypopharynx	10	18.9
	Larynx	9	17
	Other	2	3.8
cT stage	0	3	5.6
	1	5	9.4
	2	11	20.8
	3	23	43.4
	4	11	20.8
cN stage	0	5	9.4
	1	5	9.4
	2	11	20.8
	2a	2	3.7
	2b	9	17
	2c	10	18.9
	3	11	20.8
cTNM stage	III	10	18.9
	IVa	32	60.3
	IVb	11	20.8

The 400 MHZ ^1^H NMR CPMG median spectra from the preCHT and postCHT groups are shown in Fig.S1.

### Distinction between the preCHT and postCHT samples using OPLS-DA

**Fig. 1:**
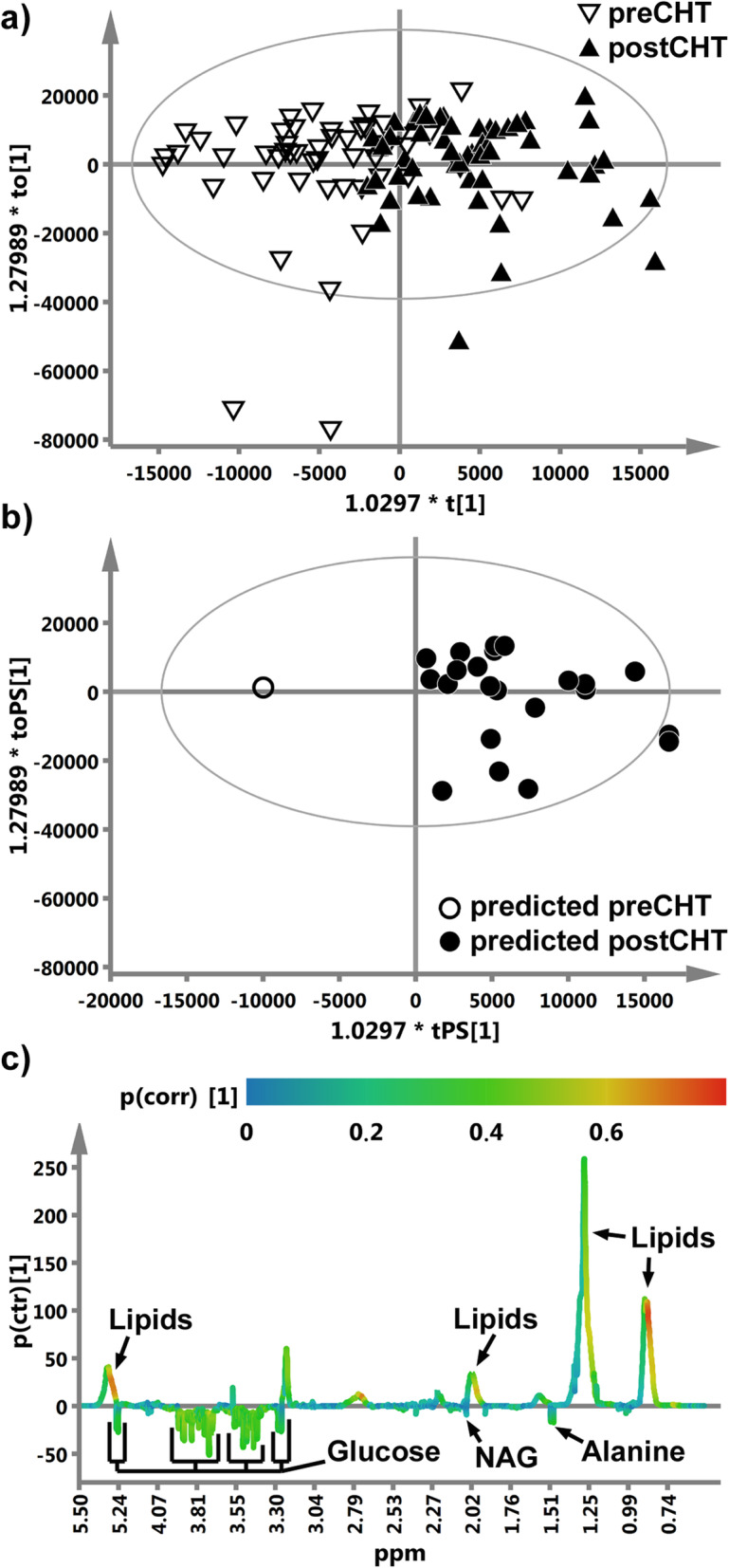
**a** The R2X scaled (the distances in the plot correspond to the explained variation) score plot obtained from the OPLS-DA analysis of the 1H CPMG NMR spectra of the serum samples from the LA-HNSCC patients before (preCHT, ) and after (postCHT, ) induction chemotherapy. **b** The R2X scaled predicted (tPS) score plot from the OPLS-DA analysis. The predicted samples from the external test set are correctly classified. **c** The OPLS-DA s-line plot indicating the metabolites that differentiate the preCHT and postCHT groups. The correlation of the particular metabolites towards a segregation between the groups (p(corr)) is assessed according to the associated color bar

The results from the OPLS-DA analysis are presented in Fig. 1. The OPLS-DA scores plot (Fig. 1a) shows a fair separation between the pre- and postCHT classes, with a small number of the mixing samples. The metabolites important for a class separation (lipids, alanine and glucose) are identified based on the s-line plot (Fig. 1c). The summary of the OPLS-DA analysis is presented in Table 2. The results of the validation procedure of the OPLS-DA model are presented in Fig. 1b (the predicted (tPS) scores plot for the external test set), Fig. S2 (the permutation plot) and Table 2 (the misclassification table for the external test set and cv-ANOVA).

**Table 2:** The results from the OPLS-DA and statistical analyses

OPLS-DA model diagnostics					
Predictive component			R2X	R2Y	Q2
			0.0995	0.477	0.28
Orthogonal components			R2X(o)		
			0.548		
			0.107		
cv-ANOVA *p* value			0.0001		
**Misclassification table**					
	Members		Correct		
preCHT	1		100%		
postCHT	23		100%		
**List of the important metabolites from the OPLS-DA model**					
Name		ppm	p(corr)	p value	Median ratio [%]
Metabolites increased after induction chemotherapy					
1	Lipids	0.9	0.76	< 0.000	12.73
		1.3	0.57	< 0.000	18.58
		5.3	0.69	< 0.000	17.38
Metabolites decreased after induction chemotherapy					
2	Alanine	1.48	0.33	0.0008	14.21
3	NAG	2.07	0.12	0.0002	9.03
4	Glucose	3.24	0.31	0.04	9.4
		3.42	0.43	0.042	8.11
		3.44	0.48	0.038	8.46
		3.51	0.43	0.036	9.54
		3.56	0.46	0.04	9.33
		3.72	0.55	0.039	9.59
		3.76	0.43	0.031	8.3
		3.83	0.39	0.019	8.57
		3.9	0.48	0.028	9.34
		5.2	0.32	0.025	9.57

R2X — an amount of variation in the data that is correlated to class separation; R2Y — a fraction of the class membership (Y) variation modeled using the data matrix (X), this parameter tells how good is the separation between two classes; R2X(o) — an amount of variation in the data that is uncorrelated (orthogonal) to the class separation; Q2 — a predictive ability of the OPLS-DA model; p(corr) — describes reliability of a variable, the closer to one the better; *p* value – from the WSR test, Median ratio — shows the between class differences in the peak integrals and is calculated as: 100 - (lower median)/(higher median)*100. NAG — N-acetyl-glycoprotein.

### Analysis of the mixing samples – investigation of demographic and clinical factors

**Fig. 2:**
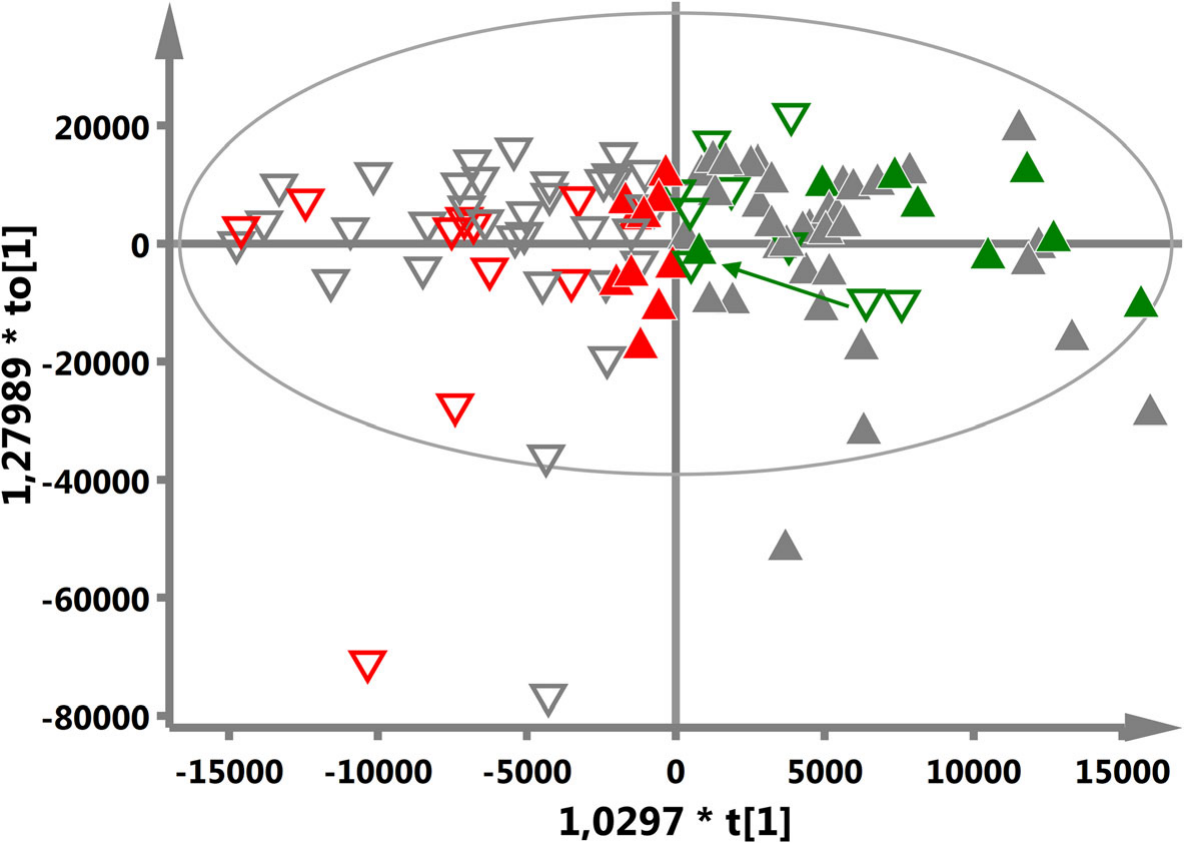
The modified Fig. 1a with the colored mixing cases. The preCHT samples are denoted as the empty triangles: - the nonmixing patients,  - the mixing patients starting the treatment with a metabolic profile similar to that of the nonmixing group before the treatment and characterized by a weaker metabolic response than the nonmixing ones, and - the patients starting the treatment with a metabolic profile similar to the Grey patients after the treatment and characterized by a weaker metabolic response than the nonmixing ones. The corresponding postCHT samples are denoted as the full color triangles: , ,  – the colors have the same meaning as for the preCHT samples. Within the mixing group, except for one patient indicated by the green arrow directed from right to left, the trajectories of the metabolic changes are directed from left (preCHT) to right (postCHT), as expected

The OPLS-DA model is able to distinguish the preCHT and postCHT samples, although the separation is not very distinct due to the cloud of the mixing samples seen in the OPLS-DA scores plot center (Fig. 1a). These mixing samples are shown in Fig. 2, a modified version of Fig. 1a, as two groups marked in red and green (if for a particular patient a postCHT sample is identified as a mixing one, the corresponding preCHT sample from this patient is also colored), whereas the non-mixing ones are greyed out (the Grey group). We identified 10 postCHT cases shifted to the left side of the plot, towards the negative values (the area occupied by the preCHT samples), and 9 preCHT cases shifted to the right side, towards the positive values (the area occupied by the postCHT samples). The mixing postCHT samples and their corresponding preCHT samples shifted to the left side of the plot are colored in red (the Red group), while the mixing preCHT samples and their corresponding postCHT samples shifted to the right side of the plot are colored in green (the Green group). Such visualization is helpful to trace the trajectories of the iCHT induced changes in the serum metabolic profiles. As seen, for both Green and Red groups the weaker metabolic response, measured as a distance between the adequate preCHT-postCHT t [1] coordinates, is observed than for the Grey, non-mixing group. This distance is the largest for the Grey group (KWA with the *p*-values of 0.027 and 0.034 for the comparison with the Red and Green ones) indicating the largest amplitude of the metabolic alterations in the grey patients. Moreover, except for one green patient (marked in Fig. 2 by the arrow), the trajectories are correct, i.e. directed from left (preCHT) to right (postCHT).

**Fig. 3:**
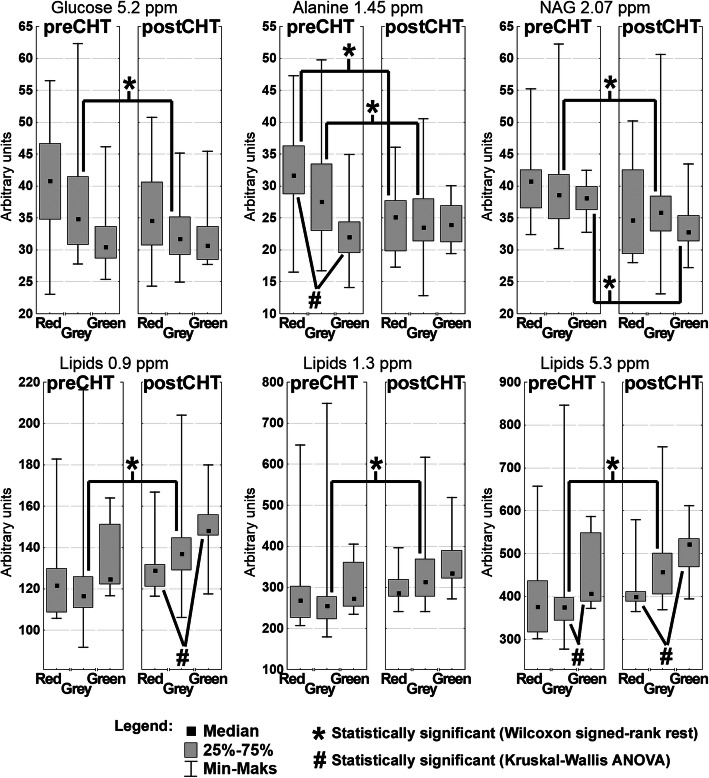
The box plot representation of the relative changes in the metabolites identified by OPLS-DA as important to differentiate the preCHT and postCHT samples. The results are presented separately for the mixing (Red, Green) and non-mixing (Grey) cases shown in Fig. 3

The box plot representation of the relative changes in the metabolites identified by OPLS-DA and the WSR test as important to differentiate the preCHT and postCHT samples in the mixing and non-mixing patients is presented in Fig. 3. The lipid signals are significantly increased after iCHT only in the non-mixing patients (*p* < 0.000, the Grey group, WSR). The Green patients start and end the treatment with the higher lipid levels as compared to the values detected for the other groups: the preCHT signal at 5.3 ppm is significantly higher (*p* = 0.04, KWA) than for the grey, non-mixing patients, whereas the postCHT lipid signals at 0.9 and 5.3 ppm are significantly higher than in the Red group (*p* = 0.011 and 0.011 respectively, KWA), however the treatment-related increase in this group is negligible. Similarly, glucose is significantly decreased after iCHT only in the non-mixing patients (*p* = 0.03, WSR). The decrease of alanine is significant in the Grey (*p* < 0.000, WSR) and Red (*p* = 0.03, WSR) groups, while the NAG is significantly decreased in the Grey (*p* = 0.005, WSR) and Green (*p* = 0.04, WSR) ones.

The baseline demographic and clinical status of the mixing patients was carefully investigated. The Green patients had significantly lower initial nodal (cN) stage when compared to the Red and the non-mixing (Grey) ones (KWA with the *p*-values of 0.029 and 0.047, respectively). There were no significant differences in the cT and cTNM stages, tumor sites, sex, age. The treatment related factors were also evaluated, showing no significant differences in the number of the iCHT cycles, overall treatment time (OTT) as well as utilization of the particular iCHT scheme (TPF/PF) between the Grey, Red and Green subgroups. Assessment of the iCHT induced toxicity revealed mostly the grade 1 or 2 (low-grade) toxicity according to the CTCAE. The most common low-grade hematological adverse events were: anemia, leukopenia, thrombocytopenia and neutropenia affecting 55, 34, 23 and 15% of the patients, respectively. The high grade (grade 3 or 4) adverse events were observed in 26% of the patients (neutropenia), 15% of the patients (leukopenia) and 4% of the patients (thrombocytopenia). The intensification of the side effects were at the same levels (KWA the *p*-values close to 1) within the subgroups.

### Influence of baseline demographic and clinical factors on the metabolic profile

The pretreatment lipids at 0.9, 1.3 and 5.3 ppm showed a weak (SCR R = -0.39, − 0.35 and − 0.36, respectively) inverse correlation with the initial TNM stage, while the pretreatment alanine correlated weakly with the initial nodal stage (R = 0.37). Furthermore, the preCHT levels of glucose and alanine were significantly (MWU *p*-values of 0.009 and 0.001, respectively) higher in males (M) compared to females (F), and thus in TPF compared to PF due to the different M/F ratios in these groups (there were fewer women treated with TPF than with PF, although the difference was not statistically significant (*p* = 0.15, MWU)).

**Fig. 4:**
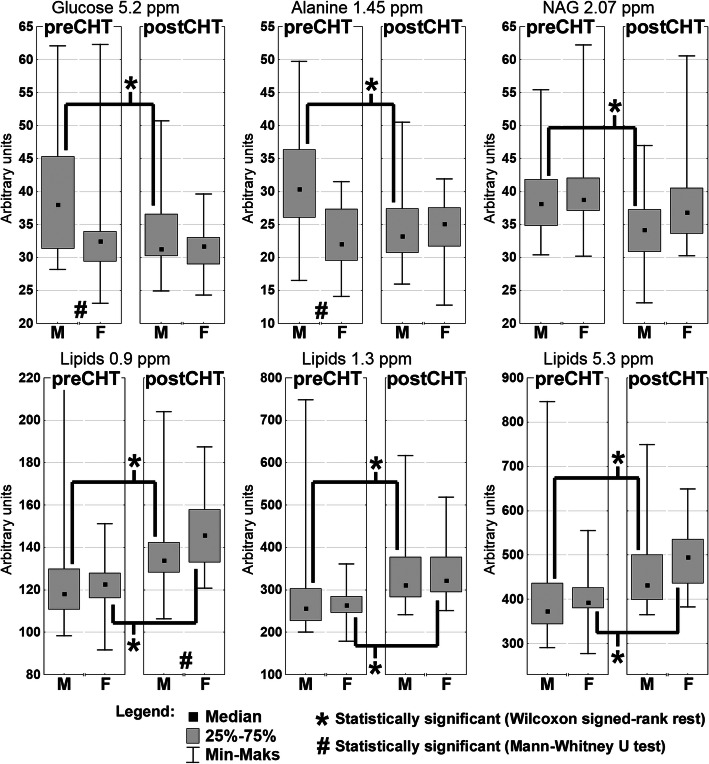
The box plot representation of the relative changes in the metabolites identified by OPLS-DA as important to differentiate the preCHT and postCHT samples. The results are presented separately for males (M) and females (F). The decrease of glucose and alanine in M results from the increased pre-treatment levels of these metabolites

The box plot representation of the relative changes in the metabolites identified by OPLS-DA and the WSR test as important to differentiate the preCHT and postCHT samples in M and F is presented in Fig. 4. It is clearly visible that only in M the levels of glucose and alanine are lower after iCHT. There are no differences in the glucose and alanine levels between M and F when comparing the postCHT samples.

### Correlation of the tumor regression after iCHT with the metabolic profile

In our study, most of the patients (87%) were the significant responders (as defined in Assessment of treatment response to induction chemotherapy), however, the degree of tumor shrinkage varied among the cases. The values of the radiologically evaluated tumor volumes, the percentages of the primary tumor regression and the initial and post-treatment clinical staging are provided in Table S3. Although the percentage of the tumor regression was higher in the Grey group compared to Red and Green, the difference was below the statistical significance. The percentage of the patients without the postCHT primary tumor and the nodal downstaging were markedly higher in Red (20%) and Green (22%) compared to Grey (8.8%), however, due to the much larger size of the Grey group than the Red and Green groups, this cannot be the basis for a statistical inference.

In order to evaluate whether the magnitude of the postCHT changes in the analyzed metabolites is correlated with a clinically or radiologically measured response to the treatment (as defined in Assessment of treatment response to induction chemotherapy), the SRC coefficients were calculated. The magnitude of the metabolic alterations was expressed as the difference between the preCHT and postCHT levels of the metabolites. The correlations were calculated for the whole group as well as separately, for the subgroups stratified according to the sex-related and metabolic responses (Grey, Red, Green). The obtained results (only with a statistical significance, i.e. with *p* < 0.05 and R > 3) are listed in Table 3.

**Table 3:** Results of the Spearman’s correlation between the clinically and radiologically measured response to the treatment and the difference between the preCHT and postCHT metabolites

Response to the iCHT	Correlation with the difference between the preCHT- postCHT metabolites					
	All patients	Stratified according to metabolic response			Sex stratified	
		Grey	Red	Green	M	F
cT-yT	Lip 1.3 ppm (R = -0.31) ↑ NAG (R = 0.33) ↓	Lip 0.9 ppm (R = -0.38) ↑ Lip 1.3 ppm (R = -0.46) ↑	–	Alanine (R = 0.7) ↓ NAG (R = 0.73) ↓	Lip 0.9 ppm (R = -0.55) ↑ Lip 1.3 ppm (R = -0.57) ↑ Lip 5.3 ppm (R = -0.47) ↑ NAG (R = 0.41) ↓	–
cN-yN	–	–	–	–	–	–
Primary tumor regression [%]	–	–	–	Alanine (R = 0.77) ↓	LIP 0.9 ppm (R = -0.45) ↑ Lip 1.3 ppm (R = -0.40) ↑	–

Legend: only statistically significant (*p* < 0.05, R > 0.3) values are presented. The arrows denote the increase/decrease of the particular metabolites, which is correlated with the tumor downstaging/shrinkage.

For the whole studied group, the clinically observed regression of the primary tumor (cT-yT) is weakly correlated with the postCHT increase of the lipid signals (R = -0.31, SRC) and the decrease of NAG (R = 0.33, SRC). In M this correlation is stronger (|R| ~ 0.4–0.57, SRC), while in F it is absent. The increase in lipids is also correlated with cT-yT in the Grey group (R = -0.38 and − 0.46, SRC), while in the Green group, the cT-yT correlates with the postCHT decrease of NAG (R = 0.73, SRC) and alanine (R = 0.7, SRC) only. The percentage of the primary tumor regression calculated based on the radiological scans is worse explained by the metabolic profile showing only the correlation with the postCHT increase in lipids (M) (R = -0.45 and − 0.4, SRC) and the alanine decrease (Green) (R = 0.77, SRC). The clinically observed regression of the nodal stage, cN-yN, shows no correlation with the important metabolites. Furthermore, no metabolic correlations between the response to the treatment in the Red group as well as in F is observed.

## Discussion

In our previous works we identified the metabolic changes in the blood serum related to the toxicity of radio- and chemoradiotherapy in the HNSCC patients [26, 27], however, the potential influence of induction chemotherapy was not assessed. In the present work we examine the serum metabolic profiles acquired before the induction chemotherapy (preCHT) and within a week before the start of a sequential radio- or chemoradiotherapy (postCHT) and analyze the metabolic changes in correlation with the conditions related to patient (age, sex), treatment (OTT, iCHT regimen, toxicity) and oncological disease (the TNM re-stage and volumetric radiological remission after treatment).

The already gathered “omic” results suggest that to expand our understanding of the iCHT effects as well as of the possible mechanisms of the toxicity induced by chemoradiotherapy and of a subsequent radiation therapy new studies should focus on the molecular mechanisms and biochemical pathways [26–28]. As reveals from our present study, the NMR-based metabolomics sheds some light on the metabolic impact and efficacy of induction chemotherapy. To the best of our knowledge, this is the first NMR based metabolomics approach to the identification of the biomarkers of induction chemotherapy in HNSCC.

### OPLS-DA mixing serum samples – the measure of response to iCHT

The OPLS-DA model with two orthogonal components successfully separated the preCHT and postCHT serum samples (Fig. 1a) as well as was able to predict the sample class from the external test group (Fig. 1b). However, there were 19 identified mixing samples located in the origin of the OPLS-DA scores plot (Fig. 1a). These mixing – Red and Green samples (with the corresponding pre/postCHT samples, 19 pairs) – were carefully checked for any clinical, demographic or treatment related conditions that may affect their metabolic profiles. The Green patients had significantly lower initial nodal stage (cN), and presumably this is a reason of their spectra to be shifted to the right in the OPLS-DA scores plot (the postCHT direction). This statement can be supported by the fact that in the study group the pretreatment lipids are inversely correlated with the initial TNM stage, while the pretreatment alanine correlates with the initial nodal stage. The Green patients had the higher levels of the lipid compounds as well as the lower level of alanine before the treatment when compared to others (Fig. 3). The iCHT-induced metabolic changes in the Green group show the correct (except for one patient) trajectory, i.e. from left to right (Fig. 2), however the response is significantly weaker than in the Grey group. The median percentage of the primary tumor regression is also lower in this group (71.8%) compared to Grey (87%), although without a statistical significance (Table S3).

In the Red subgroup, a weaker (compared to Grey) metabolic response to iCHT was also observed with only a significant decrease in the alanine postCHT level (Fig. 3) and the median percentage of the primary tumor regression of 76.4% (Table S3). However, contrary to the Green group, these patients do not differ significantly at baseline (preCHT) from the Grey group. Their glucose, alanine, NAG and lipids levels are similar to those for Grey and they do not present any differences in sex, age and the initial tumor stage.

### S-line plot – the metabolites important for discrimination between the preCHT and postCHT classes

The metabolites important for discrimination between the preCHT and postCHT classes were identified based on the OPLS-DA s-line plot (Fig. 1c). The characteristic metabolic changes for the postCHT samples are the increased lipid signals with the simultaneous decreases in alanine, glucose and NAG.

The effect of chemotherapy on the serum lipids has been studied for at least three decades [20, 29–32], mainly in breast cancer [33, 34]. To the best of our knowledge no such studies has been conducted on HNSCC patients, despite the fact that the lipid profile is altered in this malignancy [35] as well as the main chemotherapeutic agents for HNSCC (cisplatin, docetaxel and 5-fluorouracil) are reported to induce dyslipidemia [30, 32, 33]. There is no consensus in the literature about the character of the chemotherapy-induced changes in the lipid profile. However, an increase of triglycerides (TG) and low-density lipoprotein (LDL) cholesterol with a simultaneous reduction of high-density lipoprotein (HDL) cholesterol is often reported in breast cancer [29, 33, 34], wherein an increase in TG could be a potential biomarker of effective chemotherapy [29]. In colorectal cancer chemotherapy induces an increase in total cholesterol, TG, HDL and lowers the levels of LDL [31]. In the patients who favorably responded to the treatment the increased levels of the blood phospholipids were detected [20] and those with a significant chemotherapy induced HDL elevation exhibit a better 3-year disease free survival and 7-year overall survival than those without [31]. In other types of cancers an increase of total cholesterol and LDL cholesterol was also observed in the patients with a significant response to the treatment [29].

The standard NMR acquisition protocol for the measurement of the serum lipid signals (diffusion edited) and the NOESY protocol provide information about the mobile unsaturated lipids (the lipid signal at 5.3 ppm) as well as the total cholesterol (the lipid signals at 0.9 and 1.3 ppm), however without an ability to identify the particular lipoprotein subclasses, i.e. VLDL (very low density lipoprotein), LDL, HDL, etc. [36]. Nevertheless, these NMR experiments are suitable to study a metabolic response to iCHT.

Our results show a close relationship between the increase of serum lipids and a favorable response to iCHT – the postCHT increase in the lipid signals correlates with the clinical stage reduction of the primary tumor (Table 3). Such relation is not, however, observed in F as well as in the patients with a weaker metabolic response to iCHT (the Red and Green groups). In the Red group with the lowest increase of lipids after iCHT one patient discontinued chemotherapy due to myocardial infarction, one patient had a complete pathological response and eight patients ended iCHT with a partial regression. The median degree of the tumor regression in this group was slightly lower as compared to the patients who had significantly elevated lipids (Table S3). Also, in the Green group the increase of the lipid signals was insignificant, but the green patients showed the lowest median percentage of the primary tumor regression, however, without a statistical significance. For the Grey group the appropriate regression percentage was 87.01%. In the whole study group 7 patients (6 women, 1 man) showed no postCHT reduction of the clinical tumor and nodal staging (cT = yT and cN = yN), in addition two of them showed no radiological percentage of the tumor regression after iCHT (Table S3). However, there was no specific metabolic fingerprint differentiating them from others (even when the non-responding female patients where compared to those who favorably responded).

Another metabolic effect of induction chemotherapy is lowering of the levels of glucose and alanine, two metabolites which blood levels are significantly positively correlated with each other [37]. However, their reduction was observed only in the male patients and is due to the increased pretreatment levels of these metabolites as compared to the F group.

### Possible explanation of the sex-related metabolic changes in the post iCHT HNSCC patients

The detailed investigation of the available literature data gives few possible explanations of these sex-related differences: 1) Fasting blood glucose among males is found to be higher than in females [38]; 2) Impaired fasting glucose in pre-diabetes and diabetes is more prevalent in men compared to women [39]; 3) Glucose metabolism changes during the development and progression of oral and tongue squamous cell carcinomas – the serum glucose is elevated in oral cancer patients (compared to controls) and is remarkably increased in patients with a late stage disease than in those with an early stage [40]. In our study group, there were 3 confirmed diabetic patients (only the male ones, one with a type-1 diabetes; no information about the pre-diabetic status in the remaining patients), however their preCHT glucose and alanine levels were not higher as compared to the remaining male patients. In addition, after excluding the diabetics, the difference in the preCHT glucose and alanine levels between the M and F groups as well as the postCHT drop of glucose and alanine in M were still significant. Considering that men are more reluctant to go to the doctor [41], thus their disease lasts longer (is more advanced, i.e. more men with HNSCC present the nodal stages 2 and 3 as well as the TNM stage IV than women) [42], the metabolic alterations due to cancer may be more pronounced. It seems to be reflected in the structure of the study group where more men than women presented higher cN stage (28.3% of the male patients compared to 5.6% of the female patients presented cN3 stage and 14.3% of men compared to 33.4% of women presented cN stage of 0 or 1), with a difference close to a statistical significance (*p* = 0.061, MWU). In consequence, although the influence of sex and diabetic status cannot be neglected, it seems likely that the elevated preCHT glucose and alanine levels in M could be due to the severity of the disease. On the other hand, the decrease of glucose and alanine in the male patients is not correlated with the clinical or radiological response to the treatment (Table 3). However, such correlation is observed in one of the subgroups with an insignificant postCHT increase in lipids – in the Green group the decrease in alanine is significantly correlated with a favorable response to iCHT (Table 3). This is particularly surprising, because the green patients started the treatment with the lowest level of alanine.

Furthermore, glucose and alanine are the biologically correlated metabolites, i.e. alanine is one of the major substrates for gluconeogenesis during the increased demand for energy. Both these metabolites are strongly related to the patient’s nutritional status and their decreased levels in the blood serum correlate with the weight loss during the anticancer treatment [27]. In the study group, despite the higher nodal stage, in M the levels of BMI and prealbumin are higher than in F (*p* = 0.022 and 0.003 respectively, MWU). However, contrary to the NMR detected metabolites, BMI, prealbumin and albumin do not seem to be affected by iCHT, showing no differences between the preCHT and postCHT samples for both M and F (p ≅ 0.5, WSR). Thus, we can assume that the observed metabolic alterations are probably not due to the differences in the nutritional status.

### Inflammation marker vs. sex

NAG is an NMR marker of an inflammatory state [43] and it correlates well with the level of C-reactive protein (a clinical marker of inflammation) [26, 27], however in the study group such correlation was observed only for M (*p* < 0.05, R = 0.57 and 0.33 for preCHT and postCHT samples respectively, SRC). Despite the more prevalent higher N stage in the male patients, no differences were observed in the preCHT NAG between the M and F groups. An inflammation reduction was observed in both sexes, however, such effect was significant only in M (*p* = 0.001, WSR). Similarly to glucose and alanine, it may, however, be related to the initial tumor stage. Furthermore, the decrease of NAG after iCHT correlates with a favorable response to the treatment, although not in the F group (Table 3). We are not able to find the explanation for the different response of the inflammatory marker between M and F.

### Summary

NMR-based metabolomics can stratify the patients according to their response to iCHT. Further studies on a larger scale accounting for the sex and the clinical and metabolic factors are warranted.

### The study limitations

The findings of this study have to be seen in light of some limitations already mentioned earlier. The main is the small sizes of the studied groups. The importance of this limitation is all the more important as the groups are heterogeneous in terms of sex, which turns out to influence the observed effects. Moreover, the percentages of the patients in the Red, Green and Grey groups differ, which makes the statistical inference difficult. The remedy is increasing the groups – it will also significantly facilitate the NMR detection of the metabolites of low intensity in the serum NMR spectra, which in turn will help to determine the metabolic paths responsible for the observed effects. Undoubtedly, further studies should be performed on a larger scale and accounting for sex and the clinical and metabolic factors.

## Conclusions

The NMR-based metabolomic study of the serum spectra revealed that iCHT induces the marked changes in the LA-HNSCC patients’ metabolic profiles. The molecular response to iCHT involves an increase of the serum lipids which is accompanied, however only in the males, by the simultaneous decreases of alanine, glucose and N-acetyl-glycoprotein (NAG). Furthermore, in M, the iCHT induced changes in the lipid signals and NAG significantly correlate with the primary tumor regression.

The metabolomic approach makes it also possible to stratify the patients according to their response to iCHT. In the weaker responding group two sub-groups were detected: the first one with a significantly lower initial nodal stage and the second one showing no differences in the initial clinical and metabolic statuses. These effects are sex dependent. Further studies on a larger scale accounting for the sex and the clinical and metabolic factors are warranted.

## Supplementary Information

**Additional file 1: Table S1.** NMR pulse sequence parameters; **Table S2.** - Characteristics of the external test group; **Fig. S1.** The 400 MHZ 1H NMR CPMG median spectra from the preCHT and postCHT groups; **Fig. S2.** Result of permutation testing of the obtained OPLS-DA model; **Table S3.** Radiologically and clinically measured tumor regression.

## Data Availability

The datasets generated during and/or analyzed during the current study are available from the corresponding author on a reasonable request.

## Abbreviations

CHRT: chemoradiotherapy
cN, cT: nodal (cN) and primary tumor (cT) stage before induction chemotherapy
CPMG: Carr-Purcell-Meiboom-Gill
CTCAE: common terminology criteria for adverse events
DIFF: diffusion edited
ECOG: Eastern Cooperative Oncology Group
F: female
HDL: high density lipoprotein
HPV: human papilloma virus
iCHT: induction chemotherapy
JRES: J-resolved
KWA: Kruskal-Wallis ANOVA
LA-HNSCC: locally advanced head and neck squamous cell carcinoma
LDL: low density lipoprotein
M: male
MWU: Mann-Whitney U test
NAG: N-acetyl-glycoprotein
NMR: nuclear magnetic resonance
NOESY: nuclear overhauser effect spectroscopy
OPLS-DA: orthogonal partial least squares discriminant analysis
OTT: overall treatment time
PC: chemotherapy schedule based on paclitaxel and carboplatin
PF: chemotherapy schedule based on cisplatin and 5-fluorouracil
ppm: parts per million
postCHT: after induction chemotherapy
preCHT: before induction chemotherapy
Q2: a predictive ability of the OPLS-DA model
R2X: an amount of variation in the data that is correlated to class separation
R2X(o): an amount of variation in the data that is uncorrelated (orthogonal) to the class separation
R2Y: a fraction of the class membership (Y) variation modeled using the data matrix (X)
RT: radiotherapy
SRC: Spearman’s rank correlation
TG: triglycerides
TNM: classification of malignant tumors
TPF: chemotherapy schedule based on docetaxel, cisplatin and 5-fluorouracil
tPS: predicted OPLS-DA score
VLDL: very low density lipoprotein
WHO: World Health Organization
WSR: Wilcoxon signed rank test.
