# Supplementary material for: Molecular response to induction chemotherapy and its correlation with treatment outcome in head and neck cancer patients by means of NMR-based metabolomics
Source: BMC Cancer. 2021 Apr 15;21:410. doi: 10.1186/s12885-021-08137-4 (PMC8048324; doi:10.1186/s12885-021-08137-4)
Supplement: Supplementary file 1 — Additional file 1: Table S1. NMR pulse sequence parameters; Table S2. - Characteristics of the external test group; Fig. S1. The 400 MHZ 1H NMR CPMG median spectra from the preCHT and postCHT groups; Fig. S2. Result of permutation testing of the obtained OPLS-DA model; Table S3. Radiologically and clinically measured tumor regression. [file 12885_2021_8137_MOESM1_ESM.docx]

Supplementary material for:

**Molecular response to induction chemotherapy and its correlation with treatment outcome in head and neck cancer patients by means of NMR-based metabolomics**

Łukasz Boguszewicz^1*^, Agata Bieleń^2^, Jarosław Dawid Jarczewski^3^, Mateusz Ciszek^1^, Agnieszka Skorupa^1^, Krzysztof Składowski^2^, Maria Sokół^1^

^1^ Department of Medical Physics, Maria Sklodowska-Curie National Research Institute of Oncology, Gliwice Branch

**^2^** 1^st^ Radiation and Clinical Oncology Department, Maria Sklodowska-Curie National Research Institute of Oncology, Gliwice Branch

* corresponding author: [Lukasz.boguszewicz@io.gliwice.pl](mailto:Lukasz.boguszewicz@io.gliwice.pl)

**Acknowledgements**

This work is supported by the Institutional Grand from the Medical University of Silesia, Katowice, Poland, No KNW- 1 -131/N/6/K.

**The characteristics of the acquired spectra as well as the pulse sequence parameters**

- NOESY (Nuclear Overhauser Effect Spectroscopy) – an overview of all types of molecules.
- CPMG (Carr-Purcell-Meiboom-Gill) - information on only low molecular weight metabolites.
- DIFF (diffusion edited) - mainly macromolecular signals.
- Two dimensional (2D) JRES (J-resolved) – visualization of scalar couplings and improved metabolite identiﬁcation.

**Table S1. NMR pulse sequence parameters.**

| Pulse program | **NOESYGPPR1D** | **CPMGPR1D** | **LEDBPGPPR2S1D** | **JRESGPPRQF** |
| --- | --- | --- | --- | --- |
| **TD** | 65536 | 65536 | 65536 | 8192 |
| **SW** [ppm] | 30 | 20 | 30 | 16.62 |
| **AQ** [sec] | 2.73 | 4.09 | 2.73 | 0.62 |
| **D1** [sec] | 4 | 4 | 4 | 2 |
| **D8** [sec] | 0.01 | - | - | - |
| **D16** [sec] | - | - | 0.0002 | 0.0002 |
| **D20** [sec] | - | 0.0003 | 0.12 | - |
| **D21** [sec] | - | - | 0.005 | - |
| **DS** | 4 | 4 | 4 | 16 |
| **L4** | - | 126 | - | - |
| **NS** | 32 | 64 | 64 | 1 |
| **DELTA1** [sec] | - | - | 0.11572488 | - |
| **DELTA2** [sec] | - | - | 0.004172 | - |

**The characteristics of the external test group**

**Table S2. The characteristics of the external test group.**

| Baseline patient characteristics of the main studied group | | | |
| --- | --- | --- | --- |
|  |  | No. | % |
| Age, years | |  |  |
|  | Median | 57 | |
|  | Range | 41-62 | |
| Sex | |  |  |
|  | Male | 23 |  |
|  | Female | 1 |  |
| Primary tumor site | |  |  |
|  | Oropharynx | 9 |  |
|  | Nasopharynx | 2 |  |
|  | Hypopharynx | 2 |  |
|  | Larynx | 7 |  |
|  | Other | 4 |  |
| cT stage | |  |  |
|  | 2 | 4 |  |
|  | 3 | 10 |  |
|  | 4 | 10 |  |
| cN stage | |  |  |
|  | 0 | 5 |  |
|  | 1 | 4 |  |
|  | 2 | 5 |  |
|  | 2a | 2 |  |
|  | 2b | 3 |  |
|  | 2c | 3 |  |
|  | 3 | 2 |  |
| cTNM stage | |  |  |
|  | III | 4 |  |
|  | IVa | 18 |  |
|  | IVb | 2 |  |

The second (external test) group of patients was used to test the multivariate model and consisted of 23 men and 1 woman, all Caucasians, at the median age of 56 (41–67 years) with cancer diagnosed in the following anatomical structures: nasopharynx (2 patients), oropharynx (9 patients), hypopharynx (2 patients), larynx (7 patients) and other (4 patients). All were staged to TNM IV (20 patients) and III (4 patients). There were no patients with metastases (M0 = 100%). All patients were HPV negative. The external test group was separated from the main group due to the lack of preCHT (23 patients) or postCHT (1 patient) blood sample.

**The 400 MHZ ^1^H NMR CPMG median spectra from the preCHT and postCHT groups**
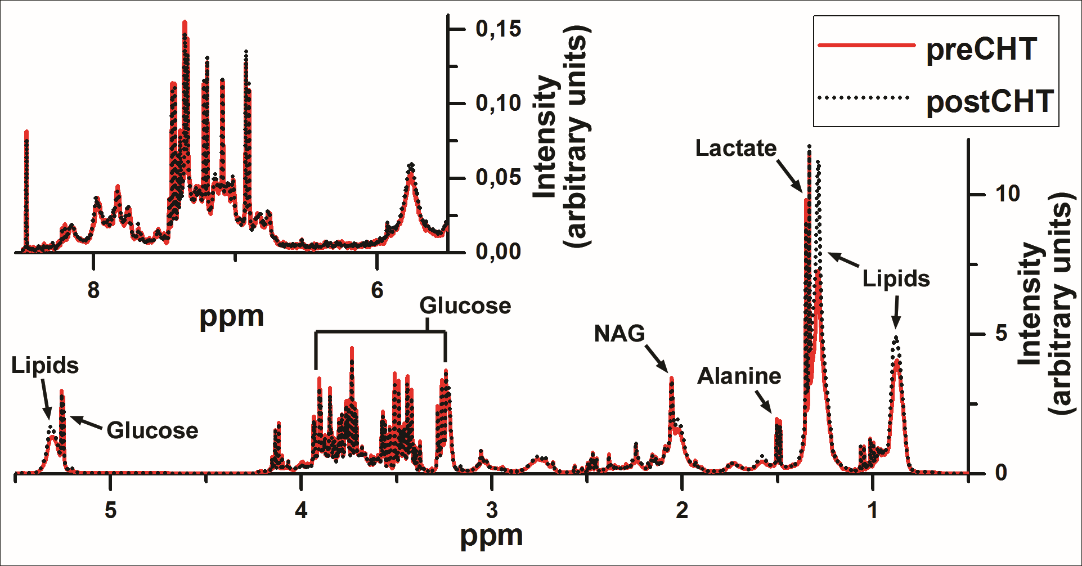


Figure S1. Median 1H-CPMG spectra of the serum samples obtained from the preCHT and postCHT groups. The main NMR detectable blood serum metabolites are indicated. The intensities of NMR resonance signals are given in arbitrary units. The aromatic region (left upper corner) is magnified 55 times compared with the aliphatic region.

**The result of permutation testing of the obtained OPLS-DA model**


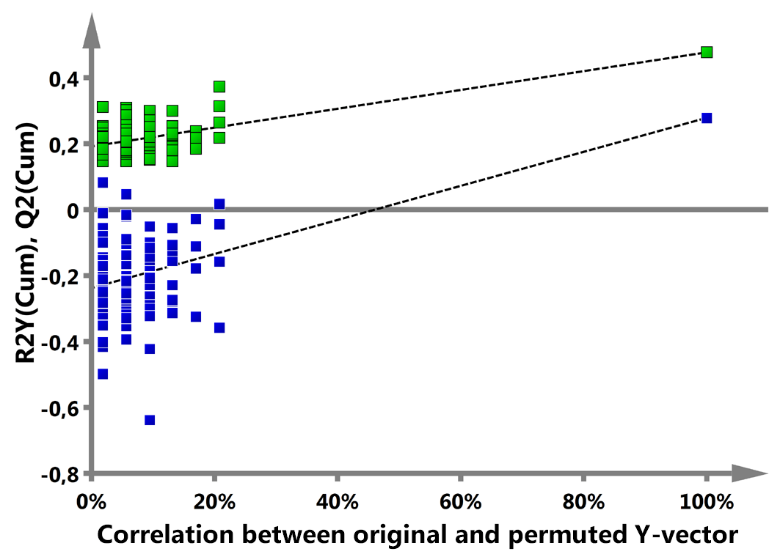


Figure S2. The permutation test for the OPLS-DA model.

**Radiologically and clinically measured tumor regression**

Table S3. The values of the radiologically evaluated tumor volumes, the percentages of the primary tumor regression as well as the initial and post-treatment clinical staging stratified for three subgroups characterized by the metabolic response to iCHT.

| Patient no. | Primary tumor size [cm^3^] | | Primary tumor regression [%] | Clinical tumor and nodal stage | | | | | Patient no. | Primary tumor size [cm^3^] | | Primary tumor regression [%] | Clinical tumor and nodal stage | | | |
| --- | --- | --- | --- | --- | --- | --- | --- | --- | --- | --- | --- | --- | --- | --- | --- | --- |
|  | preCHT | postCHT |  | Pre  CHT | | Post  CHT | | |  | preCHT | postCHT |  | Pre  CHT | | Post  CHT | |
|  |  |  |  | T | N | T | N | |  |  |  |  | T | N | T | N |
| Non-mixing patients in OPLS-DA model (GRAY) | | | | | | | | | Patients with postCHT samples shifted (RED) | | | | | | | |
| 1 | 5.53 | 0 | 100 | 4a | 2c | 0 | | 2b | 35 | 14.39 | 4.96 | 65.53 | 3 | 2 | 1 | 2 |
| 2 | - | 4.16 | - | 3 | 2c | 1 | | 2c | 36 | 76.78 | 29.81 | 61.17 | 4 | 2 | 4 | 2 |
| 3 | 42.45 | 0 | 100 | 2 | 3 | 0 | | 2b | 37 | 1 | 0 | 100 | 1 | 3 | 0 | 2b |
| 4 | 6.3 | 1.77 | 71.9 | 3 | 2b | 1 | | 0 | 38 | 10.57 | 6.55 | 38.03 | 1 | 3 | 1 | 0 |
| 5 | 1.43 | 1 | 30.01 | 3 | 0 | 2 | | 0 | 39 | 10.95 | 1.03 | 90.59 | 2 | 2b | 1 | 2b |
| 6 | 16.47 | 0 | 100 | 3 | 1 | 0 | | 0 | 40 | 1.12 | 0.98 | 12.5 | 4 | 2b | 4 | 2b |
| 7 | - | 1.21 | - | 3 | 2a | 2 | | 0 | 41 | - | - | - | 0 | 2a | 0 | 1 |
| 8 | 26.31 | 26.31 | 0 | 4 | 2 | 4 | | 2 | 42 | 21.72 | 2.77 | 87.25 | 3 | 2c | 1 | 1 |
| 9 | 9.56 | 2.84 | 70.29 | 3 | 2c | 2 | | 2c | 43 | 19.3 | 1.24 | 93.57 | 3 | 2 | 1 | 0 |
| 10 | 7.67 | 5 | 34.82 | 3 | 2b | 3 | | 2b | 44 | - | - | - | 0 | 3 | 0 | 2b |
| 11 | 15.66 | 4.67 | 70.18 | 3 | 2c | 1 | | 2c | **MIN** | **1** | **0.0** | **12.5** |  |  |  |  |
| 12 | 8.5 | 0 | 100 | 1 | 3 | 0 | | 0 | **MAX** | **76.78** | **29.81** | **100** |  |  |  |  |
| 13 | 3.59 | 1.26 | 64.9 | 4a | 2b | 1 | | 2b | **MED** | **12.67** | **2.01** | **76.39** |  |  |  |  |
| 14 | 3 | 0 | 100 | 4a | 2b | 0 | | 1 | **Number of patients without tumor downstage (cT=yT, cN=yN)** | | | | | **2**  **(20%)** | | |
| 15 | 11.26 | 11.26 | 0 | 2 | 1 | 2 | | 1 |  |  |  |  |  |  |  |  |
| 16 | 14.06 | 0.62 | 95.59 | 2 | 2c | 1 | | 2c | Patients with preCHT samples shifted (GREEN) | | | | | | | |
| 17 | - | - | - | 0 | 3 | 0 | | 2b | 45 | 6.96 | 1.21 | 82.61 | 2 | 2c | 2 | 2c |
| 18 | 34.62 | 2.48 | 92.84 | 3 | 2 | 1 | | 2 | 46 | 18.44 | 9.05 | 50.92 | 3 | 0 | 2 | 0 |
| 19 | 14.39 | 5.14 | 64.28 | 4a | 1 | 1 | | 0 | 47 | 9.8 | 3.15 | 67.86 | 2 | 2c | 1 | 1 |
| 20 | 16.43 | 0.78 | 95.25 | 3 | 3 | 1 | | 2c | 48 | 25.31 | 7.37 | 70.88 | 1 | 2 | 1 | 2 |
| 21 | 1.27 | 0 | 100 | 2 | 2 | 0 | | 0 | 49 | 8.57 | 1.38 | 83.9 | 3 | 1 | 1 | 0 |
| 22 | - | 0 | 100 | 2 | 3 | 0 | | 1 | 50 | 18.56 | 5.79 | 68.8 | 1 | 1 | 1 | 0 |
| 23 | 29.62 | 4.25 | 85.65 | 4 | 2 | 1 | | 0 | 51 | 6.63 | 0 | 100 | 3 | 2b | 0 | 2b |
| 24 | 12.94 | 0 | 100 | 3 | 2c | 0 | | 2c | 52 | 33.26 | 9.38 | 71.8 | 3 | 0 | 2 | 0 |
| 25 | - | 8.37 | - | 3 | 3 | 1 | | 2c | 53 | 10.4 | 1.12 | 89.23 | 4a | 0 | 1 | 0 |
| 26 | 29.1 | 16.71 | 42.58 | 3 | 2 | 2 | | 1 | **MIN** | **6.63** | **0** | **50.92** |  |  |  |  |
| 27 | 22.22 | 0 | 100 | 4 | 2 | 0 | | 1 | **MAX** | **33.26** | **9.38** | **100** |  |  |  |  |
| 28 | 2.58 | 0.71 | 72.48 | 3 | 0 | 1 | | 0 | **MED** | **10.4** | **3.15** | **71.8** |  |  |  |  |
| 29 | 22.23 | 9.6 | 56.82 | 2 | 2 | 1 | | 2 | **Number of patients without tumor downstage (cT=yT, cN=yN)** | | | | | **2**  **(22%)** | | |
| 30 | 12.28 | 2.19 | 82.17 | 4a | 2b | 1 | | 0 |  |  |  |  |  |  |  |  |
| 31 | 26.78 | 6.32 | 76.4 | 3 | 2c | 2 | | 2c |  | | | | | | | |
| 32 | 6.32 | 0.41 | 93.51 | 2 | 2b | 1 | | 0 |  |  |  |  |  |  |  |  |
| 33 | 7.81 | 0.9 | 88.48 | 2 | 3 | 1 | | 0 |  |  |  |  |  |  |  |  |
| 34 | 43.35 | 1.03 | 97.62 | 3 | 3 | 2 | | 2 |  |  |  |  |  |  |  |  |
| **MIN** | **1.27** | **0** | **0** |  |  |  | |  |  |  |  |  |  |  |  |  |
| **MAX** | **43.35** | **26.31** | **100** |  |  |  | |  |  |  |  |  |  |  |  |  |
| **MED** | **12.94** | **1.21** | **87.01** |  |  |  | |  |  |  |  |  |  |  |  |  |
| **Number of patients without tumor downstage (cT=yT, cN=yN)** | | | | | **3**  **(8.8%)** | | | |  |  |  |  |  |  |  |  |
